# Production of a novel laccase from *Ceratorhiza hydrophila* and assessing its potential in natural dye fixation and cytotoxicity against tumor cells

**DOI:** 10.1186/s43141-023-00473-1

**Published:** 2023-02-09

**Authors:** Yasmin M. Elsaba, Heba M. El-Hennawi, Mona M. Ibrahim, Hala R. Wehaidy

**Affiliations:** 1 Botany and Microbiology Department, Faculty of Sciences, Helwan University, Cairo, Egypt; 2 Dyeing, Printing and Textile Auxiliaries Department, National Research Centre, Dokki, Giza, Egypt; 3 Plant Biotechnology Department, National Research Centre, Dokki, Giza, Egypt; 4 Chemistry of Natural and Microbial Products Department, National Research Centre, Dokki, Giza, Egypt

**Keywords:** *Ceratorhiza hydrophila* MK387081, Flavonoid dye fixation, Laccase cytotoxicity

## Abstract

**Background:**

Flavonoid natural dyes have gained attention because they are nontoxic and eco-friendly. However, they do not work effectively with artificial fibers and require the use of mordants, which are considered as hazardous chemicals. Laccase enzyme catalyzes the oxidation of phenols, forming phenoxyl radicals that undergo a further polymerization process. So, laccase can oxidize flavonoid dyes, and it can be used instead of harmful mordants in flavonoid dye fixation on cotton fabrics. Laccases also are involved in a variety of metabolic processes, and they have anti-proliferative effects toward HepG2 and MCF-7 tumor cells.

**Results:**

Among fifteen fungal isolates, the fungus *Ceratorhiza hydrophila* isolated from the submerged plant *Myriophyllum spicatum* was selected as the most potent laccase producer. Optimization of the production medium resulted in a 9.9-fold increase in laccase productivity. The partially purified *Ceratorhiza hydrophila* laccase could successfully improve the affinity of cotton fabrics toward quercetin (flavonoid) dye with excellent color fastness properties. The partially purified laccase also showed anti-proliferative activity against HepG2 and MCF-7 tumor cells. However, high laccase concentration is required to estimate IC50.

**Conclusions:**

*Ceratorhiza hydrophila* MK387081 is an excellent laccase producer. The partially purified laccase from *Ceratorhiza hydrophila* can be used in textile dyeing and printing processes as a safer alternative to the conventional hazardous mordants. Also, it can be used in preparation of cancer treatment drugs. However, further studies are needed to investigate IC50 for both cell types at higher laccase concentrations.

## Background

Laccases (E.C.1.10.3.2) are polyphenol oxidases with copper atoms at the catalytic site and play an essential role in lignin hydrolysis [1]. They are found in a diversity of living organisms, including plants, insects, fungi, and bacteria [2–5]. Laccase catalyzes the oxidation of phenolic compounds with low- or high-molecular weights with the reduction of molecular oxygen to water [6, 7]. Fungi are considered the most important microbes for laccase production, especially those belonging to Basidiomycetes, including various genera such as *Pleurotus*, *Coriolopsis*, *Phanerochaete*, *Cerena*, *Lentinus*, and *Trametes* [8]. Laccases have potential applications in lignin degradation [9], biosensors [10], textile dyes, detoxification of polluted water, and other biotechnological processes [11]. In addition, the anticancer activities of some fungal laccases have been reported in the last years [5].

Natural dyes have gained attention because they are nontoxic and environmentally friendly as they reduce the toxicity of synthetic dye effluent. However, they do not work effectively with artificial fibers and require the use of mordants, which are metallic salts of aluminum, iron, chromium, and copper to provide good color fastness to light and washing [12]. Flavonoids (polyphenolic pigments) are natural coloring compounds found in plants, fruits, and vegetables that can be used in textile dyeing and printing. They can be oxidized by laccase enzyme to form o-quinones radicals that undergo further polymerization process which helps in fixation of dyes on textile fabrics. Since natural dyes alone have no affinity to fibers, laccase enzyme can be used instead of hazardous mordants to produce an affinity between the dye and the fiber [13].

Laccases are involved in a variety of metabolic processes, and they have anti-proliferative effects [14]. For example, laccase purified from the mushroom *Agrocybe cylindracea* had been reported to have anti-proliferative activity toward HepG2 and MCF-7 tumor cells [15]. Also, the fungal extract of *Coriolus versicolor* and *Funalia trogii* had a cytotoxic effect against HeLa cancer cell lines [16]. Laccases may play a role in cytotoxicity effect on estrogen receptor (ER)-positive breast cancer cells by preventing the binding of 17β-estradiol to the receptor (due to degradation), which is a key pathway for breast cancer cell migration, invasion, and proliferation by transmitting rapid signals inside the cells via kinases [17, 18].

The major drawback with traditional cancer treatment techniques is the inability to discriminate between normal and malignant cells. Chemotherapies and radiotherapies were among the first cancer treatment strategies, and they are still used today, although they have serious side effects. As a result, safer cancer therapy procedures have been developed, which destroy cancer cells without harming the patient’s normal cells. Laccase enzyme can destroy cancer cells without causing damage to normal cells, so it has attracted attention recently as a prospective agent for cancer treatment.

The present work is aimed at producing laccase enzyme from a novel source as this is the first study on laccase production from the fungus *Ceratorhiza hydrophila* MK387081 isolated from the submerged aquatic plant *Myriophyllum spicatum*. It also intends to assess the role of *Ceratorhiza hydrophila* MK387081 laccase enzyme as a safer alternative to the harmful chemicals used in textile natural dye fixation, as very few reports have discussed this application in the literature, and they were using commercial laccase. The produced laccase enzyme has also been evaluated as an anticancer agent against HepG2 and MCF-7 tumor cells.

## Methods

### Fungal cultures

The fungal isolates used in this study were obtained from the Mycology Lab, Botany and Microbiology Department, Faculty of Science, Helwan University, Egypt. They were isolated from soil samples from different geographical areas [19, 20] (Table 1). All isolates were maintained on potato dextrose agar (PDA) slants at 4 °C.

**Table 1:** Screening of some fungal isolates for laccase productivity

Fungal isolates	Location	Source	Laccase activity
***Cladosporium sphaerospermum***	Helwan, Egypt	Soil	**-**
***Aspergillus flavus***	Makkah, Saudi Arabia	Soil	**-**
***Aspergillus niger*** **[**19**]**	Makkah, Saudi Arabia	Soil	**-**
***Curvularia lunata***	Makkah, Saudi Arabia	Soil	**-**
***Rhizopus stolonifer***	Makkah, Saudi Arabia	Soil	**-**
***Trichoderma asperellum*** **[**19**]**	Makkah, Saudi Arabia	Soil	**+**
***Aspergillus niger***	Madina, Saudi Arabia	Soil	**-**
***Penicillium*** **sp.**	Madina, Saudi Arabia	Soil	**-**
***Ceratorhiza hydrophila*** **[**20**]**	Helwan, Egypt	*Myriophyllum spicatum* plant	**+**
***Emericella nidulans*** **[**19**]**	Madina, Saudi Arabia	Soil	**+**
***Aspergillus nidulans***	Madina, Saudi Arabia	Soil	**-**
***Trichoderma harzianum***	Beijing, China	Soil	**-**
***Aspergillus terreus***	Helwan, Egypt	Soil	**-**
***Aspergillus terreus***	Beijing, China	Soil	**-**
***Rhizopus*** **sp.**	Beijing, China	Soil	**-**

### Qualitative screening for laccase production on agar plates

Fungal isolates were screened for laccase production on plates with the following composition (g/L): 3.0 peptone, 10.0 glucose, 0.6 KH_2_PO_4_, 0.001 ZnSO_4_, 0.4 K_2_HPO_4_, 0.0005 FeSO_4_, 0.05 MnSO_4_, 0.5 MgSO_4_, 20.0 agar (pH 5.0), and 0.02% guaiacol [21]. Plates were inoculated with the fungal cultures and incubated at 30 °C for 7 days. Laccase activity was visualized on plates by the formation of reddish brown zones in the medium since laccase catalyzes the oxidative polymerization of guaiacol.

### Quantitative screening for laccase production on liquid medium

In order to choose the most potent strain for laccase production, the most potent isolates in the plate test were studied in submerged culture. The liquid medium used for screening was prepared using modified PDB medium [22] containing (g/L): 200 potato infusion and 20 glucose. Fifty milliliters of the medium was distributed in 250-mL flasks, and they were inoculated with two fungal discs (0.5 cm) from 1-week-old cultures. After 1-week of incubation at 25 °C, the cultures were collected and centrifuged at 4000 rpm, and the filtrate was used as the crude enzyme extract and analyzed for enzyme activity.

### Laccase assay

Laccase activity was investigated according to Bourbonnais et al. [23] using ABTS (2,2-azino-bis (3-ethylbenzothiazoline)-6-sulfonic acid) as a substrate.

### Determination of protein contents

The protein contents of the crude and partially purified enzyme were determined by the method of Lowry et al*.* [24].

### Optimization of laccase production by *C. hydrophila*

Various parameters that affect the enzyme productivity by the most potent laccase producer, *Ceratorhiza hydrophila* MK387081 [20], were optimized to improve the enzyme production. The optimization process was carried out by evaluating the effect of each individual parameter and then applying it in the production medium at the standardized level before optimizing the next parameter. All experiments were done in duplicates.

### Effect of incubation temperatures on laccase production

The optimum temperature for the enzyme production was investigated by incubating the production medium at different incubation temperatures (15, 25, and 30 °C) for 1 week. The culture filtrate was used as the crude enzyme extract and analyzed for enzyme activity to determine the best incubation temperature.

### Effect of fermentation time on laccase production

To study the effect of fermentation time on laccase production, the potent isolate was incubated in the production medium at different incubation periods (3, 7, 10, and 14 days) under static conditions.

### Effect of different carbon sources

Different carbon sources (starch, mannitol, lactose, sucrose, fructose, galactose, and cellulose) were used to replace glucose in the production media at a concentration of 1%.

### Effect of different nitrogen sources

The effect of different nitrogen sources on laccase production was carried out by supplementing the production media with different nitrogen sources (yeast extract, corn meal, malt extract, beef extract, peptone, sodium nitrite, and sodium nitrate).

### Effect of MgSO_4_ on laccase production

Different concentrations of MgSO_4_ (1.0 to 5.0 g/L) were added to the medium to determine its effect on laccase production.

### Effect of 1mM CuSO_4_ addition on laccase production

A total of 1 mM of copper sulfate was added to the media on the 1st, 3rd, 5th, 7th, and 10th day.

### Statistical analysis

Statistical analysis of data was carried out by using one-way analysis of variance (ANOVA) followed by homogenous subsets (Duncun^a^) at a confidence level of 0.05 using the Statistical Package for the Social Sciences (SPSS) version 21.

### Partial purification of *C. hydrophila* laccase

This was carried out by acetone precipitation using different concentrations of acetone (25–75%). The produced fractions were dried and assayed for laccase activity. The fraction with the highest purification fold was used for enzyme application studies.

### Extraction of flavonoid dye from *Hibiscus sabdariffa* flowers

Flowers of *Hibiscus sabdariffa* obtained from local markets were dried at 40 °C. Twenty grams grounded powder was extracted with 200 mL of water, 80% ethanol and 80% acetone separately. The extracts were heated at 100 °C for 2 h, and then, the extracts were collected after filtration using Whatman No. 1 filter paper and used for flavonoid determination.

### Determination of total flavonoids in *Hibiscus sabdariffa* flowers extracts

Aluminum-chloride colorimetric assay was used to determine the total flavonoid content in the extracts as reported by Biju et al. [25]. One milliliter of the flavonoid extract and 4 mL of distilled water were mixed together in a 10 mL volumetric flask. A total of 0.3 mL of 5% sodium nitrite was added. A total of 0.3 mL of 10% AlCl_3_.6H2O solution was added to the mixture after 5 min. Then, 1 mL of 1.0-M NaOH was added after 5 min and diluted with distilled water. Standard solutions of quercetin were prepared (20, 40, 60, 80, and 100 μ*g*/mL). The absorbance of extracts and standard solutions were measured against blank at 510 nm with a UV/visible spectrophotometer. The total flavonoid content was determined from the calibration curve and expressed as milligrams of quercetin equivalent (QE) per gram dry weight.

### Fabrics and printing paste

The fabric used is 100% cotton, purchased from local market. The printing paste was constituted of the extracted flavonoid dye and laccase enzyme at different ratios (1:1, 2:1, and 1:2) dye to enzyme. The thickener used was sodium alginate with medium viscosity. It was prepared by soaking 4 g in 100 mL water, and then, 50 mL of the emulsion was used. The total weight of the paste was 100 g. The paste pH was adjusted to 4.0 for all experiments. The printed cotton samples were heated in the oven at 50 °C for 4 h and then washed with running water. The printed fabrics were then dried and assessed for color strength (K/S) and color fastness properties [13].

### Color measurements

The color strength (K/S) was measured by reflection spectroscopy with a Hunter Lab UltraScan PRO spectrophotometer according to a standard method [26]. The color parameters L, a, and b stand for lightness, redness, and yellowness, and they are used to specify any color by the giving values.

*a* = + value the color is in the red direction, and *b* = +value the color is in the yellow direction [27, 28].

$$\text{Fixation}\; \% = \left(\text{K}/\text{S}\right)\; \text{after}\;\text{washing}/\left(\text{K}/\text{S}\right)\;\text{before}\;\text{washing}\times100$$

### Determination of fastness properties

The printed samples were washed as per the conditions specified in the test AATCC test method. The color fastness to washing, rubbing, perspiration, and light were determined according to the AATCC test methods [28].

### Cell cultures

HepG2 (hepatocellular carcinoma) and MCF-7 (breast Adenocarcinoma) were obtained from Nawah Scientific Inc. (Mokattam, Cairo, Egypt). Cells were maintained in DMEM media supplemented with 100 mg/mL of streptomycin, 100 units/mL of penicillin and 10% of heat-inactivated fetal bovine serum in humidified, and 5% (v/v) CO_2_ atmosphere at 37 °C.

### Cytotoxicity assay

The cytotoxic effect of the partially purified *Ceratorhiza hydrophila* MK387081 laccase was assayed by Nawah Scientific Inc. (Mokattam, Cairo, Egypt). Cell viability was assessed by the SRB assay.

## Results

### Laccase production

**Fig. 1:**
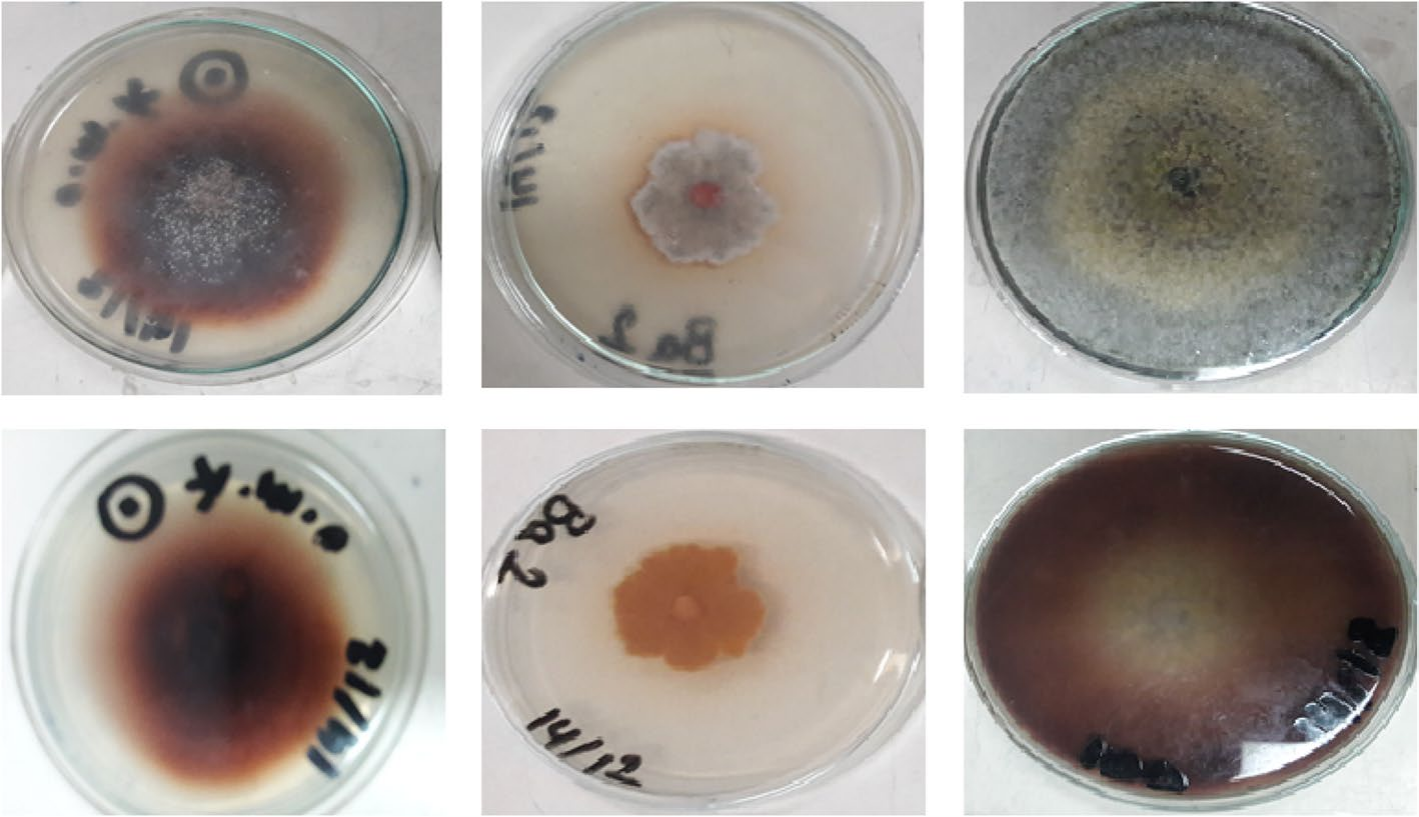
Oxidative polymerization of guaiacol to form reddish brown zones in the medium. Fungal isolates from the left to the right *C. hydrophila*, *Emericella nidulans*, and *T. asperellum*

In this study, fifteen fungal isolates were screened for their laccase activity on agar plates. As presented by Table 1, the results showed that only three isolates showed laccase activity, including *Ceratorhiza hydrophila* MK387081, *Trichoderma asperellum* MN960163, and *Emericella nidulans* as shown in Fig. 1.

The positive laccase isolates were investigated for quantitative laccase production using PDA liquid medium, and the results showed that *Ceratorhiza hydrophila* MK387081 isolated from the submerged aquatic plant *Myriophyllum spicatum* was the most potent isolate for laccase production with activity of 104 U/mL (Table 2). So, *C. hydrophila* was chosen to complete the study.

**Table 2:** Quantitative production of laccase by the potent fungal cultures

Fungal isolates	Laccase activity (UmL^−1^)
*Ceratorhiza hydrophila*	104a
*Trichoderma asperellum*	8.4b
*Emericella nidulans*	2.4c

Groups with different letters have significant differences between each other

### Optimization of laccase production by *C. hydrophila*

The optimal temperature of laccase production differs significantly from one strain to another. In the present study, the results showed that the optimum incubation temperature for laccase production was 25 °C (154 U/mL) (Table 3).

**Table 3:** Effect of temperature on *C. hydrophila* MK387081 laccase production

Temperature (°C)	Laccase activity (UmL^−1^)
15	0c
**25**	**154a**
30	104b

Groups with different letters have significant differences between each other

**Fig. 2:**
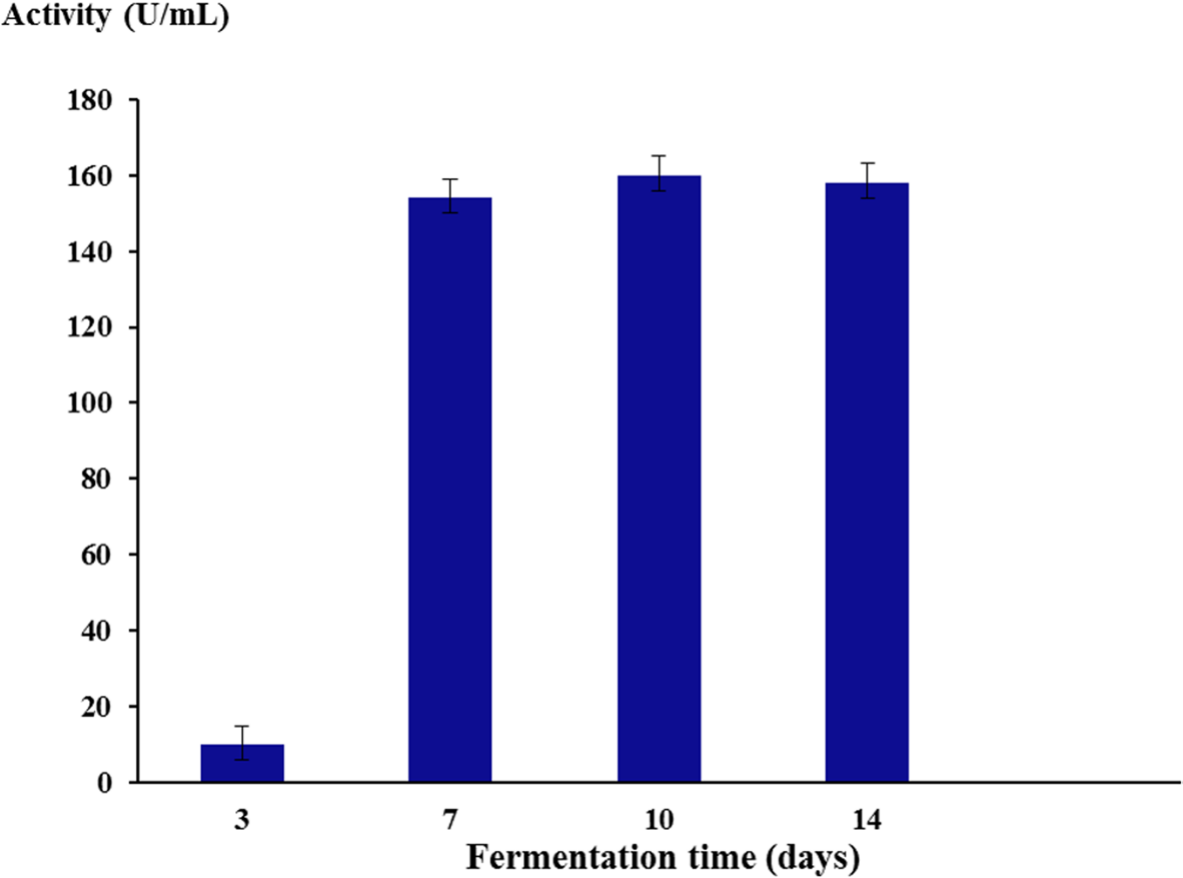
Effect of fermentation time on *C. hydrophila* MK387081 laccase production

Incubation of *C. hydrophila* MK387081 at different time intervals showed that the highest enzyme production (160 U/mL) was achieved after 10 days of incubation (Fig. 2). Enzyme production decreased after 15 days of incubation. As presented in Table 4, the maximum enzyme production was achieved with a medium containing mannitol (469 U/mL) compared to glucose used in the basal medium (160 U/mL). The highest laccase productivity (l407 U/mL) was obtained with malt extract as the best nitrogen source (Table 5). MgSO_4_ was found to have no effect on laccase production by *C. hydrophila*. Addition of 1-mM CuSO_4_ at different incubation times showed that its addition on the first day of incubation enhanced laccase enzyme production (1590 U/mL) compared to the control (1407 U/mL) (Table 6).

**Table 4:** Effect of carbon sources on *C. hydrophila* MK387081 laccase production

C-source (1%)	Laccase activity (UmL^−1^)
Glucose (control)	160b
Starch	137d
**Mannitol**	**469a**
Lactose	11h
Sucrose	58e
Fructose	20g
Galactose	153c
Cellulose	38f

Groups with different letters have significant differences between each other

**Table 5:** Effect of nitrogen sources on *C. hydrophila* MK387081 laccase production

N-source	Laccase activity (UmL^−**1**^)
Control	160b
Beef extract	56c
Yeast extract	469b
Peptone	54c
**Malt extract**	**1407a**
Corn	55c
Sod. nitrate	41d
Sodium nitrite	0e

Groups with different letters have significant differences between each other

**Table 6:** Effect of CuSO_4_ (1 mM) addition on *C. hydrophila* MK387081 laccase production

Day of CuSO_**4**_ (1 mM) addition	Laccase activity ( UmL^**-1**^)
**Control (without CuSO**_**4**_**)**	1407b
**1**	**1590a**
**3**	999c
**5**	860d
**7**	798e

Groups with different letters have significant differences between each other

## Partial purification of *C. hydrophila* laccase

The partial purification by acetone precipitation yielded 3 active fractions. The highest purification fold (1.4-fold purification compared with the crude culture filtrate) and specific activity were obtained at 50 % acetone concentration. The protein content of the fraction was estimated as 1.2 mg/mL, and the specific activity was calculated as 474.14 U/mg protein. Therefore, this fraction will be used in the upcoming studies.

### Extraction of flavonoid dye from *Hibiscus sabdariffa* flowers and dyeing of cotton fabrics

**Fig. 3:**
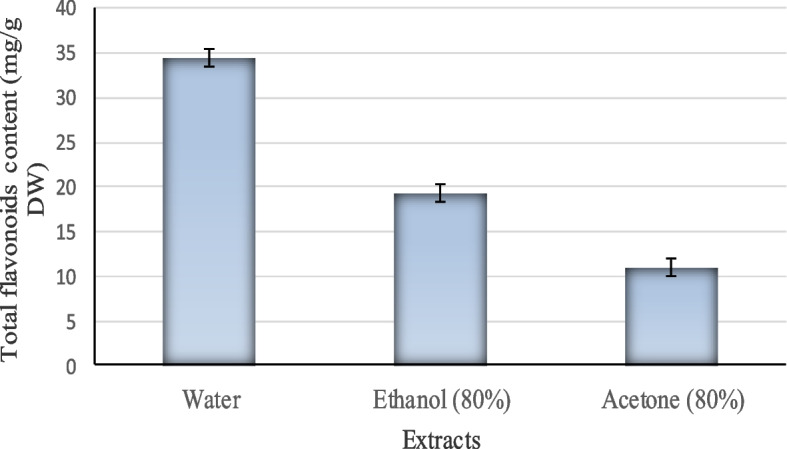
Total flavonoids content (quercetin) of *Hibiscus sabdariffa* flowers extracted by different solvents

**Fig. 4:**
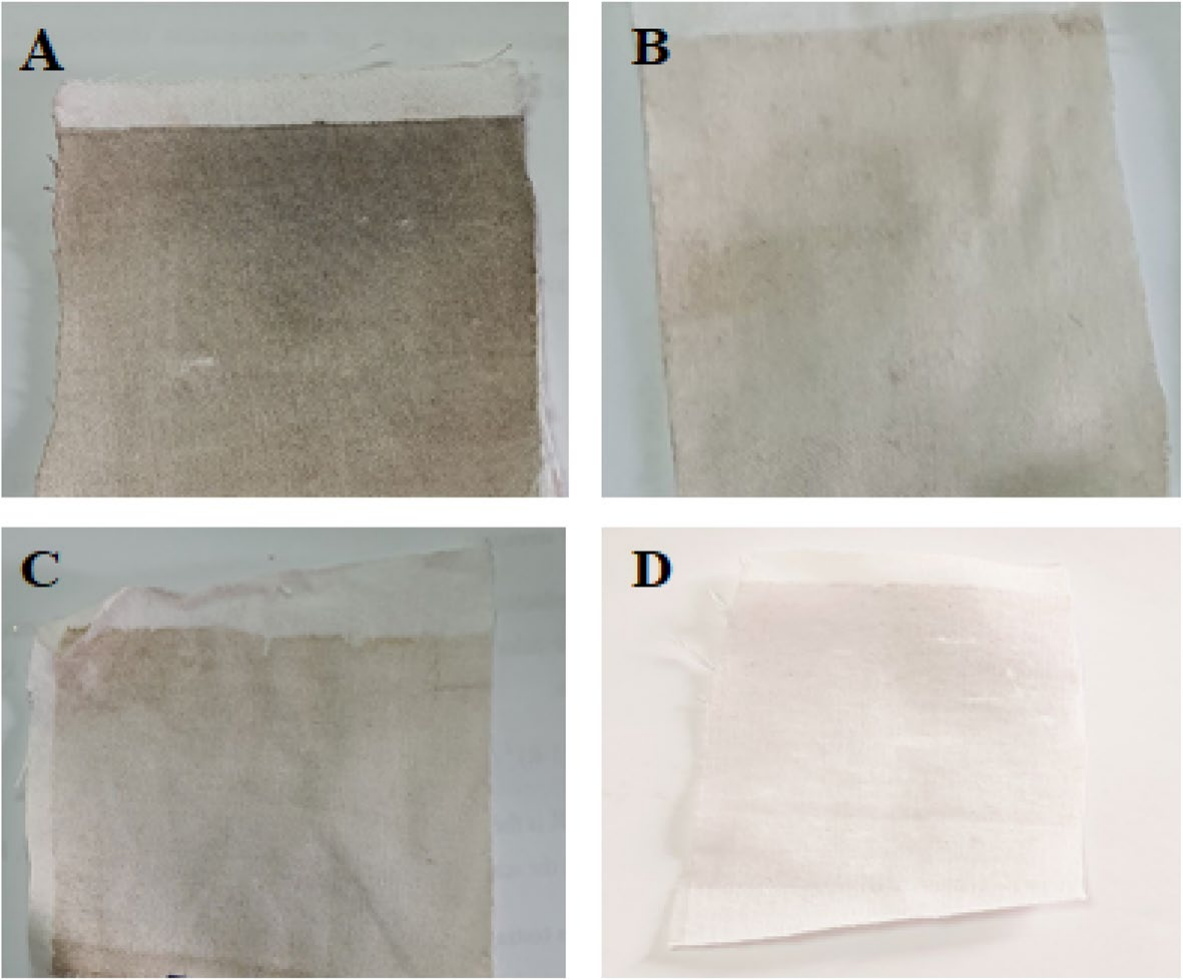
Cotton fabrics printed by quercetin extracted by different solvents. **A** Water extracted quercetin with laccase enzyme treatment. **B** Ethanol extracted quercetin with laccase enzyme treatment. **C** Acetone extracted quercetin with laccase enzyme treatment. **D** Water extracted quercetin without laccase enzyme treatment (blank)

Water extract recorded the maximum total flavonoids content (34.53.41 mg/g DW), followed by ethanol extract (19.45 mg/g DW), while acetone recorded the minimum flavonoid content (10.99 mg/g DW) (Fig. 3). The extracted flavonoid (quercetin) was used in preparing the printing paste, which has been applied to cotton fabrics. After printing, drying, fixation, and washing, the cotton fabrics were assessed for K/S and the color fastness properties. Tables 7 and 8 illustrate the effect of using water, ethanol, and acetone extracted flavonoids (quercetin) on the color strength (K/S) and color fastness properties of the printed cotton fabrics, respectively (the enzyme was mixed with dye in the ratio of 1:1). It was observed that irrespective of the solvent used for extraction, all printed fabrics acquired very good color fastness properties. However, the highest K/S value (0.94) and the highest fixation percent (88.7%) with very good color fastness properties were obtained when water was used as a solvent for quercetin extraction. Hence, *C. hydrophila* laccase could improve the affinity of cotton fabrics toward the natural dye (quercetin) (Fig. 4). As shown in Tables 9 and 10, the dye-enzyme ratio of 2:1 produced the highest K/S value, the best fastness properties, and the highest fixation percent.

**Table 7:** Effect of solvent type on color strength of cotton fabrics dyed by flavonoids

Solvent used for flavonoid extraction	K/S	***L***	***a***	***b***
Water	0.94	83.19	2.06	−2.78
Ethanol	0.75	82.8	2.01	−2.44
Acetone	0.1	81.9	2.02	−2.3

The color parameters L, a, and b stand for lightness, redness, and yellowness

**Table 8:** Effect of solvent type on color fastness properties of cotton fabrics dyed by flavonoids

Solvent used for flavonoid extraction	Rubbing fastness		Washing fastness		Perspiration fastness				Light fastness	Fixation %
	Dry	Wet	Alt.	St.	Acid		Alkaline			
					Alt.	St.	Alt	St.		
**Water**	3–4	3	4–5	4	4–5	4	4	4–5	5–6	88.7
**Ethanol**	3–4	3	3	3		3	3	3	4	75.7
**Acetone**	3	3	3	3		3	3	3	4	76.9

* St* stain, *Alt* alternate

**Table 9:** Effect of dye-laccase enzyme ratio on color strength of cotton fabrics

Dye-enzyme ratio	K/S	***L***	***a***	***b***
**Blank** (without laccase enzyme)	0.02	82.67	0.2	0.19
**1:1**	0.94	83.19	2.06	−2.78
**2:1**	1.35	79.52	2.14	1.52
**1:2**	1.28	81.9	1.36	1.27

The color parameters L, a, and b stand for lightness, redness, and yellowness

**Table 10:** Effect of dye-laccase enzyme ratio on color fastness properties of cotton fabrics dyed by flavonoids

Dye-enzyme ratio	Rubbing fastness		Washing fastness		Perspiration fastness				Light fastness	Fixation %
	Dry	Wet	Alt.	St.	Acid		Alkaline			
					Alt.	St.	Alt	St.		
**1:1**	3–4	3	4–5	4–5	3	3–4	4	3–4	5–6	96.8
**2:1**	3–4	3	4–5	4	4–5	4	4	4–5	5–6	97
**1:2**	3	2–3	4–5	4–5	3	2–3	2–3	3	4–5	88.2

*St* stain, *Alt* alternate

### Evaluation of *C. hydrophila* laccase as an antitumor agent against MCF-7 and HepG2 tumor cells

**Fig. 5:**
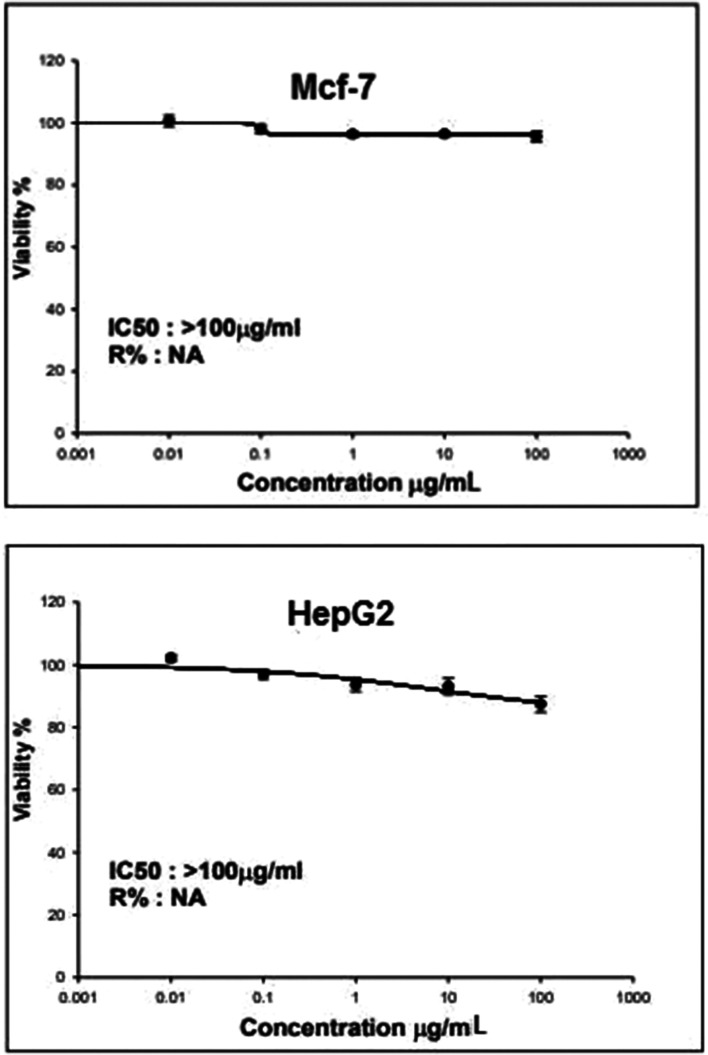
Cytotoxicity assay for MCF-7 and HepG2 tumor cells using the partially purified laccase

The partially purified laccase from *C. hydrophila* MK387081 exhibited antiproliferative activity against both MCF-7 and Hep G2 cells (Fig. 5). However, higher laccase enzyme concentration (˃ 100 μg/mL) is needed to determine IC50 in both cell types. As illustrated by Fig. 5, *C. hydrophila* laccase displayed higher antiproliferative activity toward HepG2 cells than toward MCF-7 cells.

## Discussion

To our knowledge, this is the first study of laccase production from the fungus *C. hydrophila* MK387081. *C. hydrophila* belongs to the basidiomycetous fungi, a group of fungi which are known as good laccase producers. Other basidiomycetous fungi were known as good laccase producers included *Pleurotus ostreatus*, *Ganoderma lucidum*, *Trametes pubescens*, and *Pycnoporus sanguineus* [29, 30].

Temperature influences the rate of biochemical reactions either by stimulating or suppressing enzyme production. The optimal temperature of laccase production differs significantly from one strain to another as it is a fungal-dependent factor that enhances enzyme productivity [31]. In the present study, the results showed that the optimum incubation temperature for laccase production was 25 °C. Similarly, an optimum temperature of 25 °C was reported for laccase production from *Penicillium pinophilum* (MCC 1049) [32]. Zimbardi et al. [30] detected a nearly similar optimum temperature for laccase production by *Pycnoporus sanguineus RP15* at 26 °C. On the other hand, higher optimal fermentation temperatures were recorded for laccase production from other fungal strains. For example, the maximum laccase productivity by *B. subtilis* MTCC2414 and *Chaetomium globosum* was recorded at 30 °C [33, 34]. However, the optimum temperature for laccase production by *Ganoderma leucocontextum* and *Lentinula edodes* was detected at 40 °C [35].

The maximal enzyme production was achieved after 10 days of incubation. Production decreased after 15 days of incubation, and this might be due to nutrient depletion in the medium. This observation was previously reported by Umar and Ahmed [35] for *Ganoderma leucocontextum* laccase. The high yield of laccase in a short period makes it helpful in industrial applications [36]. Many researchers obtained the maximum laccase productivity after a longer fermentation period (14, 20, and 26 days) [37–39].

Maximum enzyme production was achieved with a medium containing mannitol compared to glucose used in the basal medium. So, mannitol was the best carbon source that supported the growth and enzyme production from *C. hydrophila*. Sun et al. [40] also have observed maximum laccase productivity from *Ganoderma lucidum* strain 7071-9 in the presence of mannitol. Stajić et al. [41] have observed the highest laccase activity by *Pleurotus ostreatus* in the presence of mannitol and glucose. On the contrary, Thakkar and Bhatt [42] obtained the lowest laccase productivity when they used mannitol as a carbon source. The highest laccase productivity (l407 U/mL) was obtained with malt extract as the best nitrogen source. Hence, malt extract is essential for growth and laccase production by *C. hydrophila*. This was in agreement with Da Cunha et al. [43] who stated that malt extract was the best nitrogen source in the medium for prominent laccase production by *Phlebia floridensis*, *Phlebia brevispora*, *Phlebia radiata*, and *Phlebia fascicularia*. However, on the other hand, Keyser et al. [44] reported that fungal laccases are mainly initiated by nitrogen depletion, while Leatham and Kirk [45] reported that in some strains, laccase activity has no relation to nitrogen concentration.

MgSO_4_ was found to have no effect on laccase production by *C. hydrophila*. On the other hand, other authors reported MgSO_4_ as the main significant factor that caused a considerable increase in laccase production from *Coriolus versicolor* and *Agaricus blazei* [46, 47].

Copper has been considered a strong laccase inducer in several species, for example, *Neurospora crassa* [48], *Trametes versicolor* [49], and *Trametes trogii* [50]. Copper also affects the genetic transcription of laccase enzyme [51, 52]. However, many reports showed that copper ions had a negative effect on laccase productivity [53]. In this work, the addition of 1 mM CuSO_4_ on the first day of incubation enhanced laccase enzyme production. On the other hand, Wehaidy et al. [36] reported an increase in laccase enzyme productivity when 1 mM CuSO_4_ was added to the fermentation medium on the fifth fermentation day. However, addition of CuSO_4_ on the first fermentation day caused inhibition of enzyme productivity.

Many authors [37, 54] have observed increasing laccase activity at 1 mM CuSO_4_ concentrations and suppression of laccase productivity at higher CuSO_4_ concentrations. Since laccase is a multi-copper oxidase enzyme, the presence of copper in the medium might promote the enzyme synthesis. High concentrations of copper, on the other hand, may be toxic to microbial cells [55–57].

Therefore, the final optimized medium for laccase production by *C. hydrophila* was as follows (g/L): 200 potato infusion, mannitol 10, malt extract 10, and 1 mM CuSO_4_ incubated for 10 days at 25 °C. Thus, after medium optimization, laccase productivity (1590 U/mL) was increased by 9.9-fold compared with the basal medium (160 U/mL), and the protein content was estimated as 4.7 mg/mL.

Finally, it can be concluded that the optimum media composition for enzyme production differs according to the microorganism used. It is a fungal-dependent factor as each microorganism consumes certain components from the medium according to its nutritional requirement. So, the medium composition must be optimized for each enzyme producer.

The partial purification by acetone precipitation yielded 3 active fractions. The highest purification fold (1.4-fold purification compared with the crude culture filtrate) was obtained at 50% acetone concentration with a specific activity of 474.14 U/mg protein. Therefore, this fraction will be used in the upcoming studies.

The yield of total flavonoids mainly depends on the extraction solvent and extraction procedure. The total flavonoid contents of extracts were expressed as mg quercetin equivalent per gram dry weight. Water extract recorded the maximum total flavonoids content (34.53.41 mg/g DW). Many previous researches have been conducted on the estimation of flavonoids in *Hibiscus* [58–61].

The highest K/S value (0.94) and the highest fixation percent (88.7%) with very good color fastness properties were obtained when water was used as a solvent for quercetin dye extraction. This might be because the water extract had the maximum total quercetin content. Therefore, water-extracted quercetin will be used for printing on cotton fabrics throughout the upcoming work. It was observed that irrespective of the solvent used for extraction, all printed fabrics acquired very good color fastness properties. Hence, *C. hydrophila* laccase could improve the affinity of cotton fabrics toward the natural dye quercetin. As laccase enzyme could attack the OH group in flavonoid, forming highly reactive radicals (quinones) that perform nonenzymatic polymerization reactions to form colored high-molecular-weight compounds. The laccase reaction starts by formation of a radical cation followed by deprotonation of the hydroxyl group to give a radical which undergoes formation of quinonoid derivatives. The produced quinones are highly reactive substances that can polymerize spontaneously to form high-molecular-weight compounds or brown pigments which precipitate on the surface of cotton fabrics to form a colored film as obtained when binders were used [13]. This finding is in agreement with previous work [13, 62]. These advantages encourage using this coloration technique as a promising alternative to the conventional coloring (dyeing and printing) processes.

A dye-enzyme ratio of 2:1 produced the highest K/S value, the best color fastness properties, and the highest fixation percent. This might be because the low concentration of dye will be insufficient to saturate the enzyme active site which might delay its action. Mongkholrattanasit et al. [63] reported that the K/S value increased by increasing the concentration of dye when quercetin was used for dyeing silk fabrics. Barani and Rezaee [64] also observed an increase of K/S value of dyed wool fabrics by increasing the concentration of the natural dye extracted from *Achillea pachycephala* plant.

On the other hand, high laccase concentrations can cause a decrease in K/S of fabrics, and this might be due to the protein crowding around the enzyme which might interrupt its action. This was in agreement with El-Hennawi et al. [13] when rutin flavonoid was used with laccase enzyme for color fixation on cotton fabrics.

The partially purified laccase from *C. hydrophila* MK387081 exhibited antiproliferative activity against both MCF-7 and Hep G2 cells. However, a laccase concentration ˃ 100 μg/ml is needed to determine IC50 in both cell types. Also, laccase purified from the mushroom *Agrocybe cylindracea* was reported to have anti-proliferative activity toward Hep G2 and MCF-7 tumor cells and inhibitory activity toward HIV-1 reverse transcriptase [15].

It is well-known that all tumor cells are rich in quinones and quinone-like molecules. Laccase enzyme can destroy tumor cells by converting these molecules into toxic substances that cause apoptosis of cells [16]. The partially purified laccase extract might contain other bioactive compounds and/or peroxidase enzyme which might help laccase enzyme in its action. Rashid et al. [65] have studied the cytotoxicity effect of *F. trogii* laccase on prostate carcinoma (LNCaP) cells, and they stated that in the solvent extraction process, other bioactive compounds might be present beside laccase enzyme and might contribute to the cytotoxicity of the enzyme fractions.

*C. hydrophila* laccase displayed higher anti-proliferative activity toward HepG2 cells than toward MCF-7 cells. This was also observed for *Tricholoma mongolicum* laccase [66]. However, different results were obtained by Zhang et al. [67] as they observed that *Abortiporus biennis* laccase had lower anti-proliferative activity toward HepG2 cells than toward MCF-7 cells. Variations in anti-proliferative activity toward different tumor cell lines have been reported for numerous proteins. As an example, a 38-kDa caper protein has a 60-fold higher anti-proliferative activity toward HepG 2 cells than toward MCF-7 cells. Therefore, we can say that different tumor cells have different sensitivities to different antitumor proteins [68]. However, further studies will be conducted to demonstrate the anti-proliferative activity of *C. hydrophila* MK387081 laccase against both cell types at higher laccase concentration (˃ 100 μg/ml) and also to determine IC50.

## Conclusions

As this is the first investigation on laccase synthesis from *Ceratorhiza hydrophila* MK387081, the present study provides a novel laccase from this fungus. The partially purified *C. hydrophila* laccase could improve the affinity of cotton fabrics toward the natural dye, and it can be used as an eco-friendly substitute for the conventional harmful mordants. As *C. hydrophila* laccase attack the OH group in quercetin dye, forming highly reactive radicals that perform polymerization reactions to form high-molecular-weight compounds, forming a colored film on the cotton fabric surface. A dye-enzyme ratio of 2:1 produced the highest color strength, the best color fastness properties, and the highest fixation percent. Very few studies reported the use of commercial laccase enzyme with quercetin (purchased from companies) for textile printing. However, better fastness properties were obtained in our study. Moreover, in our study, both laccase enzyme and quercetin were produced in the lab with no costs. *C. hydrophila* MK387081 laccase exerted anti-proliferative activity against HepG2 and MCF-7 tumor cells. However, further studies are needed to learn more about the mechanism of laccase cytotoxicity and to investigate IC50 for both cell types at higher laccase concentrations.

## Data Availability

Not applicable.

## Abbreviations

*C. hydrophila*: *Ceratorhiza hydrophila*
HepG2: Hepatocellular carcinoma cell line
MCF-7: Breast cancer cell line
IC50: The half-maximal inhibitory concentration
QE: Milligrams of quercetin equivalent
